# The relationship between cyberbullying victimization and cyberbullying perpetration: The role of social responsibility

**DOI:** 10.3389/fpsyt.2022.995937

**Published:** 2022-09-09

**Authors:** Jun Zhan, Yue Yang, Rong Lian

**Affiliations:** ^1^Postdoctoral Station of Psychology, School of Psychology, Fujian Normal University, Fuzhou, China; ^2^Department of Ideology, Morality and Rule of Law, Fujian Agriculture and Forestry University, Fuzhou, China; ^3^Department of Psychology, School of Teachers' Education, Zhejiang Normal University, Jinhua, China

**Keywords:** cyberbullying victimization, cyberbullying perpetration, social responsibility, college students, moral behavior

## Abstract

Previous studies have found some influencing factors of cyberbullying. However, little is known about how cyberbullying victimization and social responsibility influence college students' cyberbullying perpetration or about the mediating and moderating mechanisms underlying these relationships. Social responsibility involves not only individuals' deep cognition and special emotional identification of social responsibility but also their firm attitude and responsible actions. The purpose of this study is to investigate the relationship between college students' cyberbullying victimization and cyberbullying perpetration and whether this relationship is moderated by social responsibility. The study sample consisted of 1,016 Chinese college students (425 males) ranging in age from 19 to 25 (mean age 22). All participants completed questionnaires on cyberbullying victimization, cyberbullying perpetration and social responsibility. The results indicated that cyberbullying victimization is positively related to cyberbullying perpetration and that this relationship is mediated by social responsibility. This study highlights the harmful impact of cyberbullying victimization on college students, more notably, the underlying mechanisms between cyberbullying victimization and cyberbullying perpetration are explored, revealing that social responsibility can reduce the promoting effect of cyberbullying victimization on cyberbullying perpetration.

## Introduction

In the era of social media, in which new media platforms and communication methods change with each passing day, with the impact of the Internet on social development, the phenomenon of cyberbullying has quietly emerged. Since 2006, incidents of cyberbullying in China have involved social ethics, legal justice, the gap between rich individuals and poor individuals, and many other aspects, gradually leading to the spread of anger on the Internet and pollution of the Internet ecological environment. Examples include the case of Peng Yu in Nanjing in 2007^1^, the case of Guo Meimei^2^ and the retrial of Nie Shubin in 2011^3^. Furthermore, in April 2022, during the fight against COVID-19, a woman in Shanghai committed suicide by leaping to her death after being abused by netizens for paying 200 yuan to express gratitude to a delivery driver. There is no doubt that cyberbullying played the most direct role in the development of these events.

Cyberbullying is typically defined as aggression that is intentionally and repeatedly carried out in an electronic context (e.g., email, blogs, instant messages, text messages) against a person who cannot easily defend him- or herself (1, 2). The experience of cyberbullying has been linked to a host of negative outcomes for both individuals and organizations (e.g., schools), including anxiety, depression, substance abuse, difficulty sleeping, increased physical symptoms, decreased performance in school, absenteeism and truancy, dropping out of school, and murder or suicide (3–6). According to the 49th Statistical Report on Internet Development in China released by the China Internet Network Information Center (CNNIC), as of December 2021, the scale of Internet users in China had reached 1.032 billion, and the Internet penetration rate had reached 73.0%. Among Internet users, college students account for approximately 20%, that is, approximately 200 million people, which is basically higher than other age groups. Therefore, Chinese college students are much more likely to suffer from and commit cyber violence than other groups. A large number of existing studies focus on the problem of cyberbullying among teenagers (7–9), but relatively few studies focus on college students. In fact, the problem of cyberbullying among college students is not only very different from that among teenagers but also increasingly more acute. The physical development and mental development of college students are not synchronized. Although the physical function of college students has become mature, their ideology, moral viewpoint and moral quality have not been developed and stabilized. College students have difficulty independently, objectively and rationally facing complex network information. From the perspective of Erikson's theory of personality development and combined with Chinese social reality, most Chinese college students still face the conflict between self-identity and role confusion (10–13). In addition, compared with teens, college students have high cultural knowledge literacy and a high ability to express themselves, and their subjective initiative and self-awareness are more prominent, which makes them more likely to join in cyber violence incidents and become “keyboard warriors” who commit cyber violence.

Some researchers have summarized the internal motivation for college students' cyberbullying. First, cyberbullying is the individual's cognitive dissonance caused by the emotional intensity of pure malicious attacks. The reality of experiencing past violence on social network platforms might induce a sense of frustration and anger, which in turn might drive individuals to engage in cyberbullying as an act of revenge. However, the relationship between cyberbullying victimization and cyberbullying perpetration among college students needs to be confirmed. According to a survey released by Chinese researchers in 2021, 21.72% of Chinese college students think they are victims of cyberbullying, 13.86% have played the role of perpetrator of cyberbullying, and 8.04% of college students think they have played the dual role of victim and perpetrator of online violence (14). “Pure emotional” cyberbullying is mainly manifested by watching and imitating inflammatory, offensive and insulting remarks to achieve a certain degree of spiritual reward and psychological compensation.

According to the general aggression model, individuals' aggression behavior is mainly affected by individual factors and environmental factors. Individual factors include personality traits, attitudes, motives, gender, beliefs, values, long-term goals, behavioral scripts, and any other consistent characteristics that the individual brings to the situation. On the other hand, environmental factors are characteristics of the environment and include aggressive cues, provocation, sources of frustration, drugs, external sanctions, and incentives. Environmental factors also include the degree to which the social situation restricts or offers an opportunity to act aggressively (7). In addition to the motive of revenge, college students' cyberbullying may be an irrational moral judgment under the impact of their immature psychological development and multiple values. Although the physiology of college students is close to maturity, their psychological development, values and personality characteristics are unstable. The contradictory psychology of conformity and originality is intertwined, which intensifies college students' experience of being blindly induced by public opinion on social network platforms to do wrong. Cyberbullying is usually caused by moral anomie (such as official corruption, tabloid scandals, abuse, or a minority student). It is easy for the broad masses of netizens to cause “justice” as an act of moral criticism because its extremism spreads widely and quickly, and reasons such as searching for pornography eventually evolve into “moral judgment” cyberbullying. In the face of such events, college students usually blindly participate in them out of simple justice motives. The original intention of justice and morality is gradually masked and distorted, and it finally evolves into irrational moral judgment in the name of moral abuse and violation of privacy.

Based on the general aggression model, whether it is a vindictive or an irrational moral judgment of online violence, individuals' aggressive behaviors are closely related to their values and perceptions. Past studies have consistently held that the tendency of moral disengagement is the most significant personality trait leading to youth cyberbullying (15, 16). Moral disengagement refers to the cognitive tendency of individuals to rationalize their own behavior in a certain way to minimize the harmfulness of their own behavior when they commit immoral behaviors (15). Specifically, the lack of a social field, the decrease in social clues and the lack of face-to-face communication between people in the network environment may lead to the lack of moral clues in the network environment, leading to the moral disengagement of college students with weakened moral awareness and lower moral standards. The positive predictive effect of moral disengagement on cyberbullying suggests that if college students have a high sense of social responsibility, which is a typical personality trait and psychological process that is the complete opposite of moral disengagement, it should theoretically be difficult to produce cognitive and behavioral tendencies toward vindictive aggression and irrational moral judgment, and vice versa. Cyberbullying is a reflection of negative psychology and emotions accumulated over time in real society, as well as a reflection of people's declining moral level, weak legal awareness and lack of social responsibility in real life. College students' social responsibility is not only a kind of internal, static state of mind, consciousness, emotional experience, or quality but also a psychological process based on the exterior, the social responsibility of dynamic action (17). College students have not only a profound cognition of social responsibility and special emotion recognition but also a resolute attitude of social responsibility and responsible action, meaning that there is an organic unity of college students' social responsibility cognition, social responsibility identification and social responsibility action. However, few empirical studies have directly focused on the actual impact of individuals' social responsibility cognition, social responsibility identification and social responsibility action on cyberbullying. If the negative prediction of the individual's social responsibility for their cyber violence is confirmed, it will provide strategic recommendations for future school and family education to eliminate cyber violence.

In conclusion, at present, there are few empirical studies on the phenomenon of cyberbullying among Chinese college students, and there is also a lack of empirical studies to directly prove the relationship between cyberbullying victimization and cyberbullying perpetration of college students, as well as the role of social responsibility in this relationship. Considering that the method of experimental research is limited by the sample size and there is no mature research paradigm for cyberbullying at present, the method of investigation is adopted in this study. Therefore, this study focuses on Chinese college students and conducts a questionnaire survey to explore the impact of individuals' social responsibility on their online violence behavior, especially the relationship between cyberbullying victimization, cyberbullying perpetration and social responsibility. In view of the findings and conclusions of previous studies, the following basic hypotheses are proposed: 1. Cyberbullying victimization can positively predict the tendency toward cyberbullying perpetration. 2. Social responsibility can negatively predict the tendency toward cyberbullying. 3. The impact of cyberbullying victimization on cyberbullying perpetration is influenced by social responsibility.

## Methods

### Participants

A total of 1,331 national college students were recruited to voluntarily participate in the survey, and the data of 115 subjects were excluded (data missing), for a questionnaire recovery rate of 90%. The remaining 1,016 subjects (425 males) were aged between 19 and 25 (mean average 22). In order to avoid the subjects filling out the questionnaire out of the social approval tendency, we did not inform the subjects of the nature of the questionnaire and the purpose of the test, and the questionnaire was conducted anonymously. After completing the survey, each subject was paid 15–20 RMB. The questionnaire was approved and tested by the Academic Ethics Committee of Fujian Agriculture and Forestry University.

### Measures

#### Cyberbullying questionnaire

The Cyberbullying Questionnaire developed by Doane et al. (18) was translated and used into Chinese in the study (18). The questionnaire contains two subscales, the cyberbullying perpetration scale and the cyberbullying victimization scale. The cyberbullying perpetration scale has 20 questions and 4 dimensions, with 6 items for malice (1. Have you sent a rude message to someone electronically? 2. Have you teased someone electronically? 3. Have you been mean to someone electronically? 4. Have you called someone mean names electronically? 5. Have you made fun of someone electronically? 6. Have you cursed at someone electronically?), three items for public humiliation (1. Have you posted an embarrassing picture of someone electronically where other people could see it? 2. Have you posted a picture of someone electronically that they did not want others to see? 3. Have you posted a picture electronically of someone doing something illegal!?), three items for deception (1. Have you pretended to be someone else while talking to someone electronically? 2. Has someone shared personal information with you electronically when you pretended to be someone else? 3. Have you lied about yourself to someone electronically?), and eight items for forced contact (1. Have you sent an unwanted pornographic picture to someone electronically? 2. Have you tried to meet someone in person that you talked to electronically who did not want to meet you in person? 3. Have you sent an unwanted sexual message to someone electronically? 4. Have you sent an unwanted nude or partially nude picture to someone electronically? 5. Have you sent a message to a person electronically that claimed you would try to find out where they live? 6. Have you tried to get information from someone you talked to electronically that they did not want to give? 7. Have you sent a message electronically to a stranger requesting sex? 8. Have you asked a stranger electronically about what they are wearing?). The cyberbullying victimization scale consists of 21 questions and four dimensions, of which five items are for malice (1. Has someone called you mean names electronically? 2. Has someone been mean to you electronically? 3. Has someone cursed at you electronically? 4. Has someone made fun of you electronically? 5. Has someone teased you electronically?), 9 items for public Humiliation (1. Has someone distributed information electronically while pretending to be you? 2. Has someone changed a picture of you in a negative way and posted it electronically? 3. Has someone written mean messages about you publicly electronically? 4. Has someone logged into your electronic account and changed your information? 5. Has someone posted a nude picture of you electronically? 6. Has someone printed out an electronic conversation you had and then showed it to others? 7. Have you completed an electronic survey that was supposed to remain private but the answers were sent to someone else? 8. Has someone logged into your electronic account and pretended to be you? 9. Has someone posted an embarrassing picture of you electronically where other people could see it?), three items for deception (1. Has someone pretended to be someone else while talking to you electronically? 2. Has someone lied about themselves to you electronically? 3. Have you shared personal information with someone electronically and then later found the person was not who you thought it was?) and four items for Unwanted Contact (1. Have you received a nude or partially nude picture that you did not want from someone you were talking to electronically? 2. Have you received a pornographic picture that you did not want from someone electronically that was not spam? 3. Have you received an unwanted sexual message from someone electronically? 4. Have you received an offensive picture electronically that was not spam?). The two subscales adopt measures ranging from 0 to 6 points for each question (0: never, 2: less than a few times a year, 3: a few times a year, 4: once or twice a month, 4: once or twice a week, 5: every day or almost every day), and with the method of a positive score, the higher the score according to the individual, the more intense the network bullying behavior tends to be. The cyberbullying perpetration subscale and cyberbullying victimization subscale had good internal consistency. The results of the chi-square test showed that the factor loadings of the two subscales were 0.76–0.96 and 0.64–0.92, meeting the statistical requirements of the valence survey (17, 19).

#### Social responsibility scale of college students

This research adopts the Social Responsibility Scale of College Students formulated by Wei (17). This instrument has 31 items and three dimensions, which are social responsibility cognition (The degree of rational understanding of social responsibility), social responsibility identification (The degree of approval for taking social responsibility) and social responsibility action (The degree of practice of specific social responsibilities). Each dimension covers political responsibility, life responsibility, learning responsibility, school responsibility and network responsibility. For example, social responsibility cognition includes the cognition of political responsibility (for example, “I know the current international situation around our country and the foreign policy adopted by our country”), life responsibility cognition (for example, “I know that in the face of danger, we should not only do what is just but also learn to do what is wise”), cognition of learning responsibility (e.g., “I have a clear learning goal and know that learning itself is also a responsibility”), recognition of school responsibility (e.g., “I know the history, current situation and development ideas of my school”) and online responsibility (e.g., “For online games, I know moderation and not addiction”). social responsibility identification includes the identification of political responsibility (for example, “I agree that it is very valuable for contemporary college students to learn Marxist theory and the system and theory of socialism with Chinese characteristics”), the identification of life responsibility (such as “I agree with the act of doing what is just, seeing what is righteous and wise, sacrificing oneself for others and so on”), the identification of learning responsibility (“I agree that knowledge is an important way to change destiny”), the identification of school responsibility (“I agree with the view that college students should take an active part in club activities at school”), and the identification of network responsibility (for example, “I agree that the state should severely punish those who spread slurs through the Internet”). Social responsibility action includes actions for political responsibility (for example, “I firmly support the Communist Party of China and firmly believe in the path, theory and system of socialism with Chinese characteristics”), actions for life responsibility (such as “I have participated in voluntary labor, blood donations, disaster relief, donations and other social welfare activities”, actions for learning responsibility (e.g., “I am able to complete assignments on time, carefully, and independently and do not cheat on exams”), actions for school responsibility (for example, “I usually pay attention to the school website news trends to understand the activities that the school held recently or that it will hold”), actions for network responsibility (such as “On the Internet, it is common to see some negative comments on national policies. I did not follow the trend of comments on the Internet that are groundless or vent dissatisfaction”). Each question is scored on a scale from 1 to 5 (1: very inconsistent, 2: relatively inconsistent, 3: intermediate, 4: relatively consistent, 5 points means very consistent). The higher the score is, the more obvious the tendency toward social responsibility. The scale has good reliability and validity. According to the measurement, the Cronbach α coefficient of the scale in this study is 0.95, and the internal consistency coefficient of each item is between 0.75 and 0.90, meeting the statistical requirements of the questionnaire survey.

### Statistical analysis

This study used standardized questionnaires to collect data online, and all data were analyzed with SPSS version 24.0 and AMOS version 24.0 for Windows. Means, SDs, the Pearson correlations among the studied variables, and the differences in independent variables such as the gender, place of birth and position of students were also reported in the primary analysis. Structural equation modeling (SEM) was employed to examine the hypothesized model. In the model, social responsibility, cyberbullying perpetration, and cyberbullying victimization were considered latent variables, and their measurement indicators were parceled using an isolated approach. The bias-corrected bootstrapping (*N* = 1,000) method was employed to estimate the confidence interval (CI) of the indirect effect of perpetration. The model was estimated by a robust maximum likelihood estimation procedure. Several fit statistics and the criteria of the model evaluation were as follows: GFI and AGFI ≥ 0.80, CFI, TLI and RFI ≥0.90, and RMSEA ≤0.08 (20).

## Results

### Primary analyses

#### Difference analysis

The independent sample *t*-test was conducted with students' gender, position, and place of birth as the independent variables and social responsibility and its dimensions, cyberbullying victimization and its dimensions, and cyberbullying perpetration and its dimensions as the dependent variables.

The *t*-test results with gender as the independent variable show (see Table 1A) that in terms of social responsibility and its dimensions, males were significantly lower than females [social responsibility: *t*_(1014)_ = −3.12, *p* < 0.01, *d* = 0.20; social responsibility cognition: *t*_(1014)_ = −3.13, *p* < 0.01, *d* = 0.20; social responsibility identification: *t*_(1014)_ = −2.03, *p* < 0.05, *d* = 0.13; social responsibility action: *t*_(1014)_ = −3.14, *p* < 0.01, *d* = 0.20]. In terms of the scores on cyberbullying perpetration and its dimensions, males were significantly higher than females in the total score of cyberbullying, malice, unwanted contact and public humiliation [cyberbullying perpetration: *t*_(1014)_ = 6.57, *p* < 0.001, *d* = 0.42; malice: *t*_(1014)_ = 7.84, *p* < 0.001, *d* = 0.49; unwanted contact: *t*_(1014)_ = 5.81, *p* < 0.001, *d* = 0.36; public humiliation: *t*_(1014)_ = 4.70, *p* < 0.001, *d* = 0.30]. In terms of the total score on cyberbullying victimization and its dimensions, males were significantly higher than females [cyberbullying victimization: *t*_(1014)_ = 5.19, *p* < 0.001, *d* = 0.33; malice: *t*_(1014)_ = 6.51, *p* < 0.001, *d* = 0.41; deception: *t*_(1014)_ = 2.77, *p* < 0.001, *d* = 0.17; unwanted contact: *t*_(1014)_ = 3.29, *p* < 0.001, *d* = 0.21; public humiliation: *t*_(1014)_ = 4.12, *p* < 0.001, *d* = 0.26].

**Table 1:** Difference analysis among variables.

**Factor**	**Gender (M** ±**SD)**			
	**Male (*N* = 425)**	**Female (*N* = 591)**	**t**	***p***
**(A) T-test for gender difference of all variables**				
Social responsibility	128.16 ± 14.337	130.82 ± 12.604	−3.123	0.002
Cognition of social responsibility	44.90 ± 5.652	45.92 ± 4.747	−3.045	0.002
Identity of social responsibility	38.27 ± 4.629	38.83 ± 4.044	−2.034	0.042
Action of social responsibility	44.99 ± 5.614	46.06 ± 5.183	−3.140	0.002
Perpetration	18.87 ± 13.545	13.49 ± 12.354	6.474	0.000
Malice	7.98 ± 5.337	5.47 ± 4.791	7.705	0.000
Unwanted contact	5.62 ± 5.934	3.63 ± 4.931	5.638	0.000
Deception	3.60 ± 2.686	3.36 ± 2.770	1.383	0.167
Public humiliation	1.68 ± 2.429	1.03 ± 1.941	4.531	0.000
Victimization	21.54 ± 14.935	16.77 ± 14.093	5.188	0.000
Malice	7.23 ± 4.900	5.25 ± 4.676	6.505	0.000
Unwanted contact	3.68 ± 3.517	2.97 ± 3.290	3.257	0.001
Deception	4.45 ± 2.793	3.97 ± 2.705	2.771	0.006
Public humiliation	6.19 ± 6.524	4.58 ± 5.805	4.041	0.000
**(B) T-test with students' place of birth difference of all variables**				
	**Place of birth (M** ± **SD)**			
**Factor**	**Rural (*****N*** = **549)**	**City (*****N*** = **467)**	**t**	***p***
Social Responsibility	128.51 ± 14.242	131.12 ± 12.235	−3.141	0.002
Cognition of social responsibility	45.00 ± 5.505	46.08 ± 4.677	−3.397	0.001
Identity of social responsibility	38.31 ± 4.610	38.93 ± 3.895	−2.324	0.020
Social responsibility action	45.20 ± 5.595	46.10 ± 5.103	−2.673	0.008
Perpetration–Malice	16.25 ± 14.031	15.14 ± 11.972	1.363	0.173
**(C) T-test for student Cadre difference of all variables**				
	**Student cadre (yes/no) (M** ±**SD)**			
**Factor**	**Student cadre (*****N*** = **607)**	**Non–student cadre (*****N*** = **409)**	**t**	***p***
Social Responsibility	130.29 ± 13.263	128.83 ± 13.603	1.702	0.089
Cognition of social responsibility	45.80 ± 5.094	45.05 ± 5.249	2.264	0.024
Social responsibility action	45.87 ± 5.287	45.23 ± 5.526	1.856	0.064
Perpetration–Deception	3.73 ± 2.785	3.05 ± 2.613	3.938	0.000
Victimization–Deception	4.32 ± 2.711	3.95 ± 2.798	2.092	0.037
Victimization–Public humiliation	5.61 ± 6.203	4.72 ± 6.074	2.266	0.024

Only results with significant differences are shown in the table.

The *t*–test results with students' place of birth as the independent variable (see Table 1B) show that the total score on social responsibility and the scores on its dimensions of rural students are significantly lower than those of urban students [social responsibility: *t*_(1014)_ = −3.10, *p* < 0.01, *d* = 0.20; social responsibility cognition: *t*_(1014)_ = −3.35, *p* < 0.01, *d* = 0.21; social responsibility identification: *t*_(1014)_ = −2.29, *p* < 0.05, *d* = 0.15; social responsibility action: *t*_(1014)_ = −2.67, *p* < 0.01, *d* = 0.17]. In terms of the score on cyberbullying perpetration and its dimensions, the malice of rural students is significantly higher than that of urban students [*t*_(1014)_ = 1.99, *p* < 0.05, *d* = 0.13].

The *t*–test results with students' position as the independent variable (see Table 1C) show that in terms of the total score on social responsibility and the scores on each dimension, the total score on social responsibility and the scores on social responsibility cognition and social responsibility action, student cadres are significantly or marginally significantly higher than nonstudent cadres [social responsibility: *t*_(1014)_ = 1.70, *p* = 0.09, *d* = 0.11; social responsibility cognition: *t*_(1014)_ = 2.26, *p* < 0.05, *d* = 0.15; social responsibility action: *t*_(1014)_ = 1.86, *p* = 0.06, *d* = 0.12]. In terms of the scores on cyberbullying perpetration and its dimensions, the deception level of student cadres is significantly higher than that of nonstudent cadres [*t*_(1014)_ = 3.89, *p* < 0.001, *d* = 0.25]. In terms of the total score on cyberbullying victimization and its dimensions, the level of deception and publicly humiliation of student cadres is significantly higher than that of nonstudent cadres [deception: *t*_(1014)_ = 2.09, *p* < 0.05, *d* = 0.13; public humiliation: *t*_(1014)_ = 2.27, *p* < 0.05, *d* = 0.14].

#### Correlation analysis

The results of the correlation analysis show that the total score on social responsibility and its various dimensions (social responsibility cognition, social responsibility action, social responsibility identification) the total score on cyberbullying perpetration and its various dimensions (malice, deception, unwanted contact, public humiliation), and the total score on cyberbullying victimization and its dimensions (malice, deception, unwanted contact, public humiliation) were all significantly negatively correlated (*ps* < 0.01). The total score on cyberbullying perpetration (malice, deception, unwanted contact and public humiliation) and its dimensions were significantly positively correlated with the total score on cyberbullying victimization and its factors (malice, deception, unwanted contact and public humiliation) (*ps* < 0.01) (see Table 2).

**Table 2:** Correlation analysis among variables.

	**Social**	**Cognition**	**Identity**	**Action**	**Perpetration**	**Perpetration**	**Perpetration-**	**Perpetration**	**Perpetration-**	**Victimization**	**Victimization-**	**Victimization-**	**Victimization-**	**Victimization-**
	**responsibility**	**of**	**of**	**of**		**-malice**	**unwanted**	**-deception**	**public**		**malice**	**unwanted**	**deception**	**public**
		**social**	**social**	**social**			**contact**		**humiliation**			**contact**		**humiliation**
		**responsibility**	**responsibility**	**responsibility**										
Social responsibility	1	0.907^**^	0.890^**^	0.908^**^	−0.639^**^	−0.565^**^	−0.378^**^	−0.587^**^	−0.560^**^	−0.543^**^	−0.423^**^	−0.396^**^	−0.463^**^	−0.523^**^
Cognition of social responsibility	0.907^**^	1	0.728^**^	0.716^**^	−0.581^**^	−0.516^**^	−0.345^**^	−0.528^**^	−0.518^**^	−0.463^**^	−0.368^**^	−0.332^**^	−0.385^**^	−0.448^**^
Identity of social responsibility	0.890^**^	0.728^**^	1	0.719^**^	−0.575^**^	−0.483^**^	−0.353^**^	−0.545^**^	−0.509^**^	−0.486^**^	−0.347^**^	−0.352^**^	−0.428^**^	−0.488^**^
Action of social responsibility	0.908^**^	0.716^**^	0.719^**^	1	−0.572^**^	−0.525^**^	−0.327^**^	−0.519^**^	−0.491^**^	−0.519^**^	−0.421^**^	−0.386^**^	−0.442^**^	−0.483^**^
Perpetration	−0.639^**^	−0.581^**^	−0.575^**^	−0.572^**^	1	0.858^**^	0.703^**^	0.915^**^	0.811^**^	0.828^**^	0.668^**^	0.625^**^	0.679^**^	0.784^**^
Perpetration -malice	−0.565^**^	−0.516^**^	−0.483^**^	−0.525^**^	0.858^**^	1	0.468^**^	0.641^**^	0.604^**^	0.744^**^	0.712^**^	0.570^**^	0.581^**^	0.629^**^
Perpetration- unwanted contact	−0.378^**^	−0.345^**^	−0.353^**^	−0.327^**^	0.703^**^	0.468^**^	1	0.576^**^	0.428^**^	0.515^**^	0.412^**^	0.468^**^	0.394^**^	0.470^**^
Perpetration-deception	−0.587^**^	−0.528^**^	−0.545^**^	−0.519^**^	0.915^**^	0.641^**^	0.576^**^	1	0.763^**^	0.748^**^	0.529^**^	0.542^**^	0.644^**^	0.760^**^
Perpetration- public humiliation	−0.560^**^	−0.518^**^	−0.509^**^	−0.491^**^	0.811^**^	0.604^**^	0.428^**^	0.763^**^	1	0.702^**^	0.492^**^	0.466^**^	0.603^**^	0.737^**^
Victimization	−0.543^**^	−0.463^**^	−0.486^**^	−0.519^**^	0.828^**^	0.744^**^	0.515^**^	0.748^**^	0.702^**^	1	0.854^**^	0.773^**^	0.824^**^	0.900^**^
Victimization- malice	−0.423^**^	−0.368^**^	−0.347^**^	−0.421^**^	0.668^**^	0.712^**^	0.412^**^	0.529^**^	0.492^**^	0.854^**^	1	0.619^**^	0.597^**^	0.632^**^
Victimization- unwanted contact	−0.396^**^	−0.332^**^	−0.352^**^	−0.386^**^	0.625^**^	0.570^**^	0.468^**^	0.542^**^	0.466^**^	0.773^**^	0.619^**^	1	0.567^**^	0.586^**^
Victimization- deception	−0.463^**^	−0.385^**^	−0.428^**^	−0.442^**^	0.679^**^	0.581^**^	0.394^**^	0.644^**^	0.603^**^	0.824^**^	0.597^**^	0.567^**^	1	0.681^**^
Victimization- public humiliation	−0.523^**^	−0.448^**^	−0.488^**^	−0.483^**^	0.784^**^	0.629^**^	0.470^**^	0.760^**^	0.737^**^	0.900^**^	0.632^**^	0.586^**^	0.681^**^	1

** represents *p* < 0.01.

### Measurement model and hypothesized model

Confirmatory factor analysis was conducted on the factors of cyberbullying perpetration, social responsibility and cyberbullying victimization, and the results are shown in Table 3. The factor loading of each factor was >0.5, indicating a good degree of fit.

**Table 3:** Parameter estimation results of structural equation model.

			**Regression weights**	**S.E**.	**C.R**.	***p***	**Standardized regression weights**	**SMC (R^2^)**
Social responsibility	←	Victimization	−0.440	0.025	−17.903	^***^	−0.588	0.346
Perpetration	←	Social responsibility	−0.276	0.024	−11.638	^***^	−0.311	0.097
Perpetration	←	Victimization	0.488	0.025	19.900	^***^	0.735	0.540
Cognition of social responsibility	←	Social responsibility	1.000				0.852	0.725
Identity of social responsibility	←	Social responsibility	0.837	0.026	32.309	^***^	0.855	0.732
Action of social responsibility	←	Social responsibility	1.029	0.033	31.616	^***^	0.840	0.706
Perpetration-malice	←	Perpetration	1.000				0.755	0.571
Perpetration-unwanted contact	←	Perpetration	1.197	0.042	28.605	^***^	0.857	0.734
Perpetration-deception	←	Perpetration	0.378	0.022	17.136	^***^	0.541	0.293
Perpetration- public humiliation	←	Perpetration	0.454	0.017	26.912	^***^	0.814	0.663
Victimization-malice	←	Victimization	0.570	0.023	24.816	^***^	0.692	0.479
Victimization-unwanted contact	←	Victimization	0.477	0.016	30.541	^***^	0.826	0.682
Victimization-deception	←	Victimization	0.325	0.014	23.746	^***^	0.697	0.486
Victimization-public humiliation	←	Victimization	1.000			^***^	0.954	0.911

*** represents *p* < 0.001.

**Figure 1:**
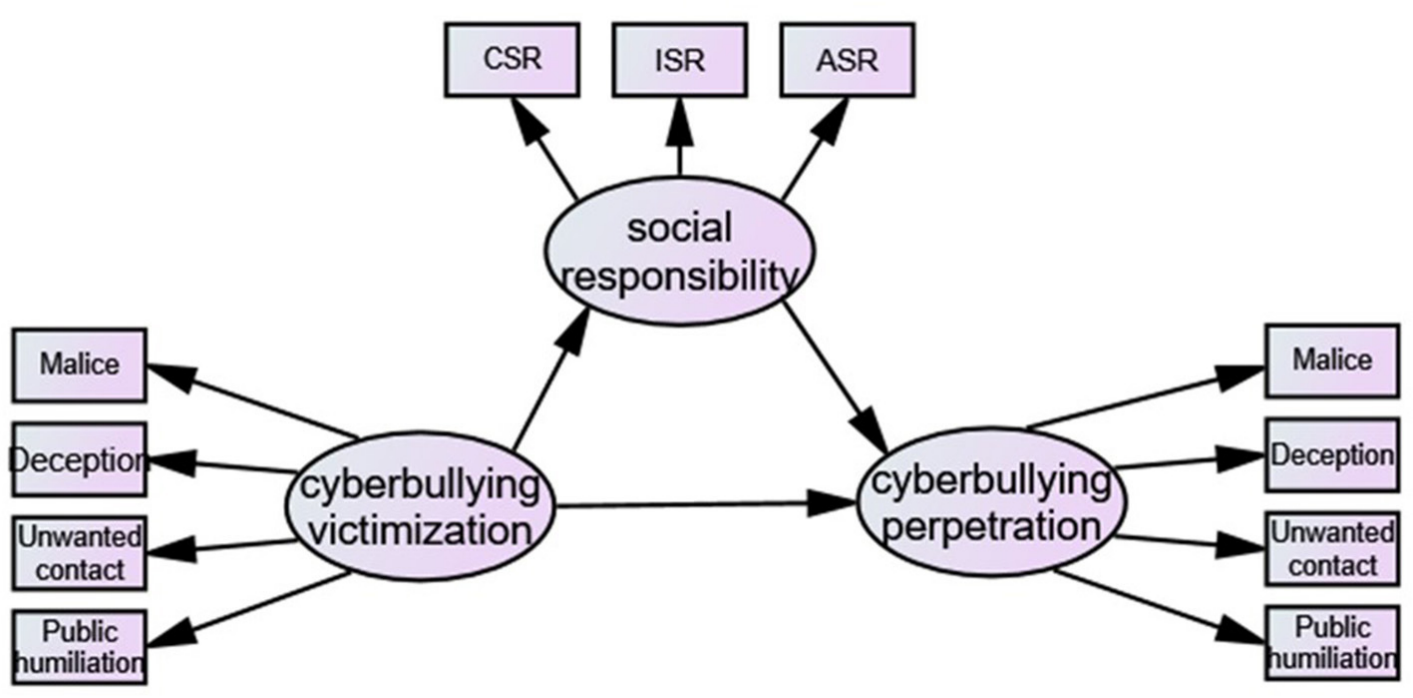
Hypothesis model of the relationship among cyberbullying victimization, cyberbullying perpetration and social responsibility.

Based on existing theories and research, this study assumes that cyberbullying victimization can directly affect cyberbullying perpetration and indirectly affect cyberbullying perpetration through social responsibility, which directly affects cyberbullying perpetration (see Figure 1).

### Structural equation model

**Figure 2:**
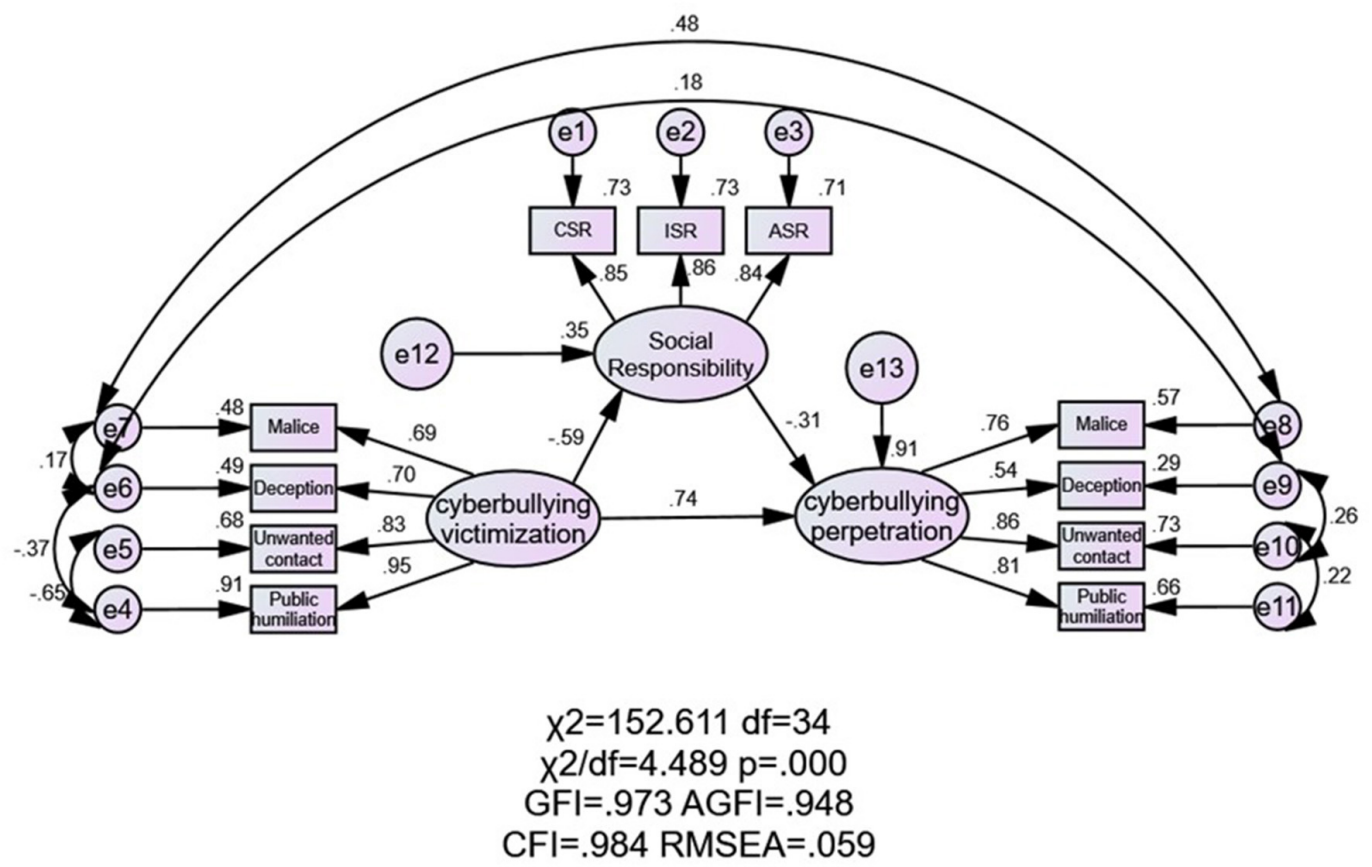
The SEM of the relationship among cyberbullying victimization, cyberbullying perpetration and social responsibility. SEM, stands for structural equation model; CSR, stands for cognition of social responsibility; ISR, stands for identification of social responsibility; ASR, stands for action of social responsibility.

Maximum likelihood (ML) estimation was used for the continuous fitting of the initial model, and modification indexes (M.I.) were comprehensively considered. According to the M.I. and theoretical basis, the factors of cyberbullying victimization and cyberbullying perpetration have potential variations (21). That is, the “malice” factor of cyberbullying victimization and cyberbullying perpetration (*M.I*. = 118.54, *r* = 0.71, *p* < 0.01) and the “deception” factor of cyberbullying victimization and cyberbullying perpetration are significantly correlated (*M.I*. = 27.65, *r* = 0.47, *p* < 0.01). The variations of the latent variables of cyberbullying victimization and cyberbullying perpetration also have variations among factors. The “malice” and “deception” factors of cyberbullying victimization (*M.I*. = 56.06, *r* = 0.62, *p* < 0.01), the “deception” and “public humiliation” factors of cyberbullying victimization (*M.I*. = 12.63, *r* = 0.59, *p* < 0.01), the “unwanted contact” and “public humiliation” factors of cyberbullying victimization (*M.I*. = 12.24, *r* = 0.68, *p* < 0.01), the “deception” and “unwanted contact” factors of cyberbullying perpetration (*M.I*. = 30.20, *r* = 0.39, *p* < 0.01), and the “unwanted contact” and “public humiliation” factors of cyberbullying perpetration (*M.I*. = 32.92, *r* = 0.43, *p* < 0.01) are significantly correlated. The final model was obtained by adding two-edged arrows representing the homogeneity of the models (Figure 2). When the data sample size is large, the chi-square test usually shows statistically significant model fit results. This study is based on a large sample (*N* > 1,000), so the chi-square test is not an important index of model fit. The fitting results show that the model is basically acceptable [χ*2* = 152.61 (*df* = 34), *p* < 0.001, *GFI* = 0.97, *CFI* = 0.98, *AGFI* = 0.95, *RMSEA* (90% *CI*) = 0.059 [0.049, 0.068], *RFI* = 0.97, *TLI* = 0.98]. It can be seen that, except for the chi-square test, the other model fitting indexes all indicate that the model has a good degree of fit. The fitting indexes and evaluation criteria are shown in Table 4.

**Table 4:** Model fitting indexes and fit criteria.

**Indices**	**χ^2^/df**	***p***	***GFI***	***CFI***	***AGFI***	***RMSEA***	***RFI***	***TLI***
Fit criteria	1~5	> 0.05	> 0.8	> 0.9	> 0.8	<0.08	> 0.90	> 0.90
The model	4.489	<0.001	0.973	0.984	0.948	0.059	0.968	0.975

Cyberbullying victimization has a negative and direct effect on social responsibility (estimates: −0.59, *SE* = 0.04, *p* < 0.01). Social responsibility has a direct negative effect on cyberbullying perpetration (estimates: −0.31, *SE* = 0.03, *p* < 0.01). Cyberbullying victimization has a positive and direct effect on cyberbullying perpetration (estimates: 0.74, *SE* = 0.02, *p* < 0.01). The standardized indirect effect is 0.18, the total effect of cyberbullying victimization on cyberbullying perpetration (ES = 0.74 +0.18 = 0.92), the direct effect accounts for 80.06%, and the indirect effect of cyberbullying victimization accounts for 19.93%. The moderated mediating effect was also tested. The bias-corrected percentile bootstrap method (1,000 repeats) was used to test the mediating effect and to observe the upper limit and lower limit of the 95% CI with bias correction. It was found that the lower limit of the indirect effect of cyberbullying victimization on cyberbullying perpetration was 0.093 and that the upper limit was 0.153. Therefore, the CI did not include 0. Part of the mediating effect through social responsibility is significant (estimates: 0.18, *SE* = 0.015, *p* < 0.001), and the mediating effect accounted for 19.93% of the total effect.

## Discussion

Cyberbullying is a typical subcultural phenomenon that is a product of the Internet era, and it does great harm to the physical and mental health of college students and the harmony and stability of society. However, it is not clear whether college students who are victims of cyberbullying also engage in more cyberbullying. We extended knowledge about this issue by examining the mediating effect of social responsibility to confirm that this factor can help account for and alter this relationship. At the same time, this study also performed a difference analysis on the indexes of students' gender, place of birth and position. The results show that compared with female college students, male college students suffer from more cyberbullying and commit more cyberbullying. Compared with urban students, rural students have more malicious cyber behaviors and lower social responsibility. Student cadres' social responsibility, online deception behavior, experience being deceived by the network and experience being publicly humiliated are significantly higher than those of other college students. In addition, a significant positive correlation between cyberbullying victimization and cyberbullying perpetration, a significant negative correlation between cyberbullying victimization and social responsibility, and a significant negative correlation between social responsibility and cyberbullying perpetration were found. Moreover, social responsibility can effectively regulate the relationship between cyberbullying victimization and cyberbullying perpetration.

### The influence of students' gender, place of birth and position on cyberbullying victimization, cyberbullying perpetration and social responsibility

Overall, men are more likely to suffer from cyberbullying and commit cyberbullying than women. At present, there is no consensus on gender differences in cyberbullying behavior. For example, some studies have found that men are more likely than women to commit cyberbullying (22) and that women are more likely to be targeted by cyberbullying (23); other studies have found no gender differences in cyberbullying (24). In this study, the fact that men are more likely to engage in impulsive behaviors such as cyberbullying than women are supported by most previous studies and may be intrinsically related to changes in testosterone levels in men (25). Research on the Internet use of Chinese college students has consistently shown that the gender differences in college students' violent video game contact, moral disengagement, sensation seeking and aggressive behavior are very significant, and the scores of male students are significantly higher than those of female students (26). Violent games often involve a considerable amount of insulting, offensive dialogue. Another survey found that the proportion of boys actively browsing pornographic websites is approximately 2 times that of girls (27). Compared with women, male college students are more likely to receive unsolicited nude photos, pornographic information and other spam emails, and even their computers will be invaded by viruses and disguised as bad information on the Internet. All these factors make male college students more likely to suffer from cyberbullying than female college students. At the same time, the malicious network behavior of rural students is obviously more frequent than that of urban students. Under the background of the urban and rural dual structure of Chinese society, rural college students are in an environment that has a large gap with their native environment. Compared with urban students, rural college students are more likely to have negative attitudes, such as an inferiority complex, social fear, and hatred of rich individuals; thus, they may be prone to vent hostile emotions and commit malicious acts on the Internet.

In addition, college students who are student cadres have more online deception behaviors, as well as more experience of being deceived and publicly humiliated on the Internet. Because of its special status, the interpersonal relationships of the student cadre group are relatively complex, and the relationship between teachers and students, the relationship between student cadres and the relationship between students and non-cadres students need to be taken into account. It is relatively easy to be misunderstood, humiliated and deceived, leading to the gradual occurrence of psychological problems among student cadres, such as interpersonal sensitivity and loneliness, which may make them tend to mask their identity and lie to others when communicating online, forming a vicious cycle of cyberbullying victimization and cyberbullying perpetration.

In the difference analysis of social responsibility, first, women scored significantly higher than men on social responsibility cognition, social responsibility identification, social responsibility action and the total score, which is consistent with many research results (28, 29). Women are believed to be emotionally rich and compassionate and to feel more responsible when making judgments and attributing responsibility. Thus, they are more likely than men to integrate themselves into the social environment and have a greater sense of responsibility toward those they relate to Gilligan (30). This suggests that women are more likely than men to feel a sense of responsibility to others or society. Second, the social responsibility cognition, social responsibility identification, social responsibility action and total social responsibility scores of rural students are significantly lower than those of urban students. However, the results are at odds with a 2014 study on the social responsibility of Chinese college students, which have shown that, there is no significant difference in the cognition, identity, action and social responsibility of college students (31). Urban culture occupies the mainstream position in contemporary Chinese society, and rural college students are in a weak position in terms of capital, especially cultural capital, which is easily ignored (32). After the transition from rural life to urban life, the disappointment of value expectations and the change in the reference group aggravate the sense of relative deprivation (33), which easily generates a sense of injustice. Several studies have consistently found that rural college students' individual or intrinsic beliefs in a just world are significantly lower than those of urban college students. That is, compared with urban college students, rural college students believe that the world is more unfair to them than to others (34), and the intensification of injustice may weaken rural college students' sense of social responsibility to a certain extent. Students who are cadres have more social responsibility than those who are not cadres, which is similar to the results of previous studies (35). As a social role, student cadres require those in this position to do their best to work seriously and responsibly and to be recognized by themselves and others. These objective requirements make them show a higher sense of social responsibility (36).

### The mediating effect of social responsibility

Consistent with our hypothesis, cyberbullying victimization and its scores on all dimensions can positively predict cyberbullying perpetration and its scores on all dimensions. In other words, the more malice, publicly shaming, deception and unwanted contact college students suffer in online communication, the more frequently they will show malice, publicly shaming, deception and unwanted contact to others. This finding can be supported by the theoretical interpretation of the general aggression model. The appearance of aggression always indicates the existence of frustration, and the existence of frustration always leads to some form of aggression. College students will have strong feelings of frustration when they encounter cyberbullying. At the same time, the general aggression model holds that individual factors and environmental factors jointly affect the cognitive process to produce aggressive behaviors, which are called input variables. The generation of aggressive behaviors is largely based on the learning, activation and application of knowledge structures related to aggression in people's memory (37). Repeated exposure to violent information stimulation will increase the aggressive component of the individual's concept, attitude, expectation, perception and behavior, which will then cause the individual's desensitization to aggression and finally increase the individual's aggressive personality, which will gradually learn and strengthen aggressive behavior in a long-term attack situation. In the absence of appropriate ways to actively cope with the negative emotional experiences of violence, individuals are more likely to engage in displaced aggression, such as bullying innocent victims online.

However, unlike previous studies that ignored potential mediating mechanisms (38–40), we innovatively explored the mediating effect of social responsibility on this relationship. As expected, social responsibility fully mediated the relationship between college students' cyberbullying victimization and cyberbullying perpetration. This is a new finding, and we are not aware of any existing study that has confirmed this mediating effect. This study also goes beyond previous studies by uncovering why cyberbullying victimization can significantly increase college students' cyberbullying perpetration. The adverse effect of cyberbullying victimization on cyberbullying perpetration is explained by college students' increased social responsibility. That is, college students who are confronted with cyberbullying are more likely to suffer from a lack of social responsibility and will neglect and destroy social harmony to justify cyberbullying, which in turn will lead them to be more likely to bully others online. Many previous studies have generally confirmed that social responsibility is closely related to prosocial behavior (41, 42). Another way to understand the findings of this study is that even if college students suffer from online violence, if they have a high sense of social responsibility, they will take more actions in line with the moral and social norms of online society. According to the activation theory of social norms, social norms do not always have an impact on behavior, and only when individuals “focus” on a social norm in a specific situation will it significantly affect individuals' behavior (43). When there is more than one social norm in a situation, the activated norm has a greater influence on behavior (44). The activation theory of social norms first proposed by Schwartz emphasizes that an individual's cooperative behavior depends on the degree of responsibility activated by environmental and individual factors (45, 46). The activation process of social norms includes three stages: the perception of the necessity of action, the determination of an effective action plan, and the determination of one's own behavioral ability to complete the action. The latter two stages are mainly related to the individual's ability, while the perception of the necessity of action is mainly related to the individual's social responsibility (47). The higher the individual's sense of social responsibility is, the easier it is to perceive the necessity of maintaining social norms when social norms are broken, not only to avoid responding to violence with violence but also to maintain social norms with a higher level of online moral words and deeds.

In addition to the mediation result, the relationship between cyberbullying victimization and social responsibility is noteworthy. To the best of our knowledge, this study is the first to confirm that cyberbullying victimization is significantly associated with college students' cyberbullying perpetration. Consistent with our hypothesis, college students who experience high cyberbullying victimization are more likely to develop a low level of social responsibility. This provides new evidence to support the view of Berkowitz (48) that prior frustrations lessen the willingness to conform to the moral norms of social responsibility (48). The aggressive inclinations arising from this frustration theoretically lead to a rejection of society's behavioral standards if these norms had been learned and to a failure to learn the standards thoroughly if they had not been acquired previously. Our finding is also consistent with previous studies that indicate that the formation process of college students' social responsibility is dependent on the college students themselves, teachers, friends, parents and other personal subjects, as well as the dormitory, class, family, school, and social groups, such as the main body participating. At the same time, the endogenous and exogenous factors work together in the processes of social responsibility cognition, identification and action, which are three important links (49, 50). For example, Lanterri (51), the director of the American Research Center for Social Responsibility, determined the influencing factors of students' social responsibility based on two aspects, self-awareness disorder and the influence of family, school and community, through 15 years of experimental research (51). Unlike these studies, we extend existing research by confirming that cyberbullying victimization is a new endogenous factor of college students themselves that can significantly decrease their social responsibility. This result highlights the importance of cyberbullying victimization in shaping college students' social responsibility and indicates that cyberbullying victimization is a new risk factor for college students' formation of social responsibility.

Taken together, the findings of this study contribute to existing research and highlight the importance of social responsibility in preventing the negative effects of cyberbullying victimization on cyber violence, and they indicate that cyberbullying victimization is a new risk factor for college students' formation of social responsibility. Furthermore, our findings may be specific to Chinese culture. China is a country where a collectivist culture deeply influenced by Confucianism prevails; this culture emphasizes the individual's sense of responsibility and contribution to society. Since antiquity, Chinese culture has had the fine tradition of “worrying before the world and enjoying the joy of the world”. Since China's reform and opening up, the social responsibility of college students has always been a matter of great importance to the state and a matter of great concern to society as a whole. Important issues are also life issues that college students must face to think deeply and actively practice. What Chinese society advocates is that social and collective interests are higher than individual interests, and social responsibility is more important than other responsibilities. As a result, in a country like China, where collectivist culture prevails and social morality is emphasized, citizens' sense of social responsibility should have a higher positive impact on their moral behavior (such as cyberbullying) than other countries that emphasize individualism. In sum, we extend previous studies by confirming the mediating effect of social responsibility on the relationship between cyberbullying victimization and cyberbullying perpetration, these results not only conducive to in-depth research on the generation of cyberbullying, but also has a practical guiding role for how to intervene and reduce cyberbullying, For example, both school education and family education should pay attention to reducing or eliminating the negative influence of the past experience of online victimization, and add training for improving the attitude, cognition and behavior of social responsibility in the moral course of college students.

### Limitations

This study has some limitations. First, the questionnaire survey and subjective reports of cyberbullying victimization, cyberbullying perpetration and social responsibility do not fully reflect the individual's actual state. It is necessary to adopt an experimental research paradigm and combine evaluation-related physiological indicators [such as heart rate, skin conductance, functional magnetic resonance imaging (fMRI)] to further study the psychological and brain mechanisms of cyberbullying. Second, this study does not exclude the influence of college students' personality and other personal traits on the research results. Past studies have consistently found that a person with an aggressive personality is more likely to commit online violence. It is necessary to take stable traits such as people's personality, mental health level and emotional regulation ability as control variables or research variables in future studies to repeatedly verify the internal relationship between cyberbullying victimization, cyberbullying perpetration and social responsibility. Third, this study did not consider the potential impact of cultural differences on the research results. Individuals in Chinese culture tend to take responsibility for other people and external phenomena, while individuals in Western culture tend to take responsibility only for themselves. Chinese culture has always stressed individual social responsibility, emphasizing that “everyone is responsible for the rise and fall of the world”. However, in Western culture, individual responsibility is self-centered, and if one is required to undertake social responsibilities and obligations, doing so is regarded as an obstacle to becoming a well-functioning person (52). Therefore, the conclusion of this study that individuals with a strong sense of social responsibility are less likely to perpetrate cyberbullying due to cyberbullying victimization may not be directly extended to the Western population.

## Conclusions

This study focuses on the relationship between cyberbullying victimization, cyberbullying perpetration and social responsibility among Chinese college students. The results show that the level of cyberbullying victimization and its dimensions can positively predict cyberbullying perpetration and its dimensions. In addition, consistent with the hypothesis, social responsibility fully mediates the relationship between college students' cyberbullying victimization and cyberbullying perpetration. Another interesting finding is that cyberbullying victimization is significantly associated with college students' cyberbullying perpetration. Therefore, this study suggests that cyberbullying victimization does not necessarily lead to cyberbullying perpetration, and the key lies in whether students have a high sense of social responsibility to regulate aggressive cognition and behavioral tendencies. That is, improving social responsibility plays an important role in reducing cyberbullying perpetration among Chinese college students. At the same time, the results also show that male college students not only carry out more cyberbullying than female college students but also suffer more cyberbullying. The social responsibility of females is significantly higher than that of males. Compared with urban students, rural students have more malicious online behaviors and less sense of social responsibility. Compared with nonstudent cadres, student cadres have more social responsibility performance, more online deception behavior, and more experience of being deceived and being publicly humiliated on the Internet.

## Data availability statement

The raw data supporting the conclusions of this article will be made available by the authors, without undue reservation.

## Ethics statement

The studies involving human participants were reviewed and approved by Ethics Committee of Fujian Agriculture and Forestry University. The patients/participants provided their written informed consent to participate in this study.

## Author contributions

JZ and YY conceived of the presented idea. RL developed the theory, performed the computations, and verified the analytical methods. RL encouraged JZ and YY to investigate a specific aspect and supervised the findings of this work. All authors discussed the results and contributed to the final manuscript.

## Funding

This work was supported by the Humanities and Social Science Project of the Ministry of Education (19YJC710100), Research on the Construction of Grassroots Teaching Organization in colleges and universities from the perspective of group dynamics (112-111420047), and the Outstanding Youth Program of Fujian Agriculture and Forestry University (XJQ201931).

## Conflict of interest

The authors declare that the research was conducted in the absence of any commercial or financial relationships that could be construed as a potential conflict of interest.

## Publisher's note

All claims expressed in this article are solely those of the authors and do not necessarily represent those of their affiliated organizations, or those of the publisher, the editors and the reviewers. Any product that may be evaluated in this article, or claim that may be made by its manufacturer, is not guaranteed or endorsed by the publisher.

## Footnotes

^1^The case of Peng Yu in Nanjing: A young man, Peng, showed kindness to an old lady who fell at a bus station, and at the request of her family, he took her to the hospital. Afterwards, she took him to court, which found that he was 40% responsible for her fall, triggering netizens to morally condemn the old woman as well as group events mocking the judge.

^2^The Guo Meimei incident: On June 21, 2011, “Guo Meimei Baby” caused netizens to criticize the Red Cross Society of China (RCSC) for flaunting her wealth on Sina Weibo.

^3^The case of the retrial of Nie Shubin: Nie Shubin, a young man in Shijiazhuang, was executed for rape and murder in 1995. Years later, Mr. Wang claimed that he was the real perpetrator of the rape and murder. On June 8, 2016, the Supreme People's Court appointed the High People's Court of Shandong Province to retry the case, which triggered netizens' discussion and dissatisfaction with China's judicial system.
